# ‘Snakes & Ladders’: factors influencing access to appropriate care for children and young people with suspected juvenile idiopathic arthritis – a qualitative study

**DOI:** 10.1186/s12969-021-00531-3

**Published:** 2021-03-23

**Authors:** Tim Rapley, Carl May, Nicola Smith, Helen E. Foster

**Affiliations:** 1 Department of Social Work, Education and Community Wellbeing, Northumbria University, NE7 7XA Newcastle upon Tyne, United Kingdom; 2 Department of Health Services Research and Policy, London School of Hygiene and Tropical Medicine, WC1H 9SH London, United Kingdom; 3 Translational and Clinical Research Institute, Newcastle University, NE2 4HH Newcastle upon Tyne, United Kingdom; 4 Population and Health Sciences Institute, Newcastle University, NE2 4HH Newcastle upon Tyne, United Kingdom

**Keywords:** Access to care, Delayed diagnosis, Juvenile idiopathic arthritis, Help‐seeking behaviour, Outcome, Primary care, Pathways of care.

## Abstract

**Background:**

Many children and young people with juvenile idiopathic arthritis (JIA) experience delay in diagnosis and access to right care. The reasons for delay are multi-factorial and influenced by patient and family, clinician and organisational factors. Our aim was to explore the experiences of care, from initial symptoms to initial referral to paediatric rheumatology.

**Methods:**

We analysed one-to-one and joint qualitative interviews with families of children with JIA (n = 36) presenting to a regional paediatric rheumatology service in the UK. We interviewed 51 family members (including mothers, fathers, patients, grandmothers and an aunt) and 10 health professionals (including orthopaedic surgeons, paediatricians, paediatric immunologist, General Practitioner and nurse) and a teacher involved in the care pathway of these JIA patients. Interviews were audio-recorded and analysed according to the standard procedures of rigorous qualitative analysis - coding, constant comparison, memoing and deviant case analysis.

**Results:**

The median age of the children was 6 years old (range 1–17), with a spread of JIA subtypes. The median reported time to first PRh MDT visit from symptom onset was 22 weeks (range 4-364 weeks). Three key factors emerged in the pathways to appropriate care: (i) the persistence of symptoms *(e.g. ‘change’ such as limp or avoidance of previously enjoyed activities)*; (ii) the persistence of parents help-seeking actions *(e.g. repeat visits to primary and hospital care with concern that their child is not ‘normal’*; iii) the experience and skills of health professionals resulting in different trajectories (*e.g. no-real-concern-at-this-point or further-investigation-is-required).* JIA was more likely to be considered amongst health practitioner if they had prior experiences of a child with JIA (moreso with a ‘protracted pathway’) or exposure to paediatric rheumatology in their training. Conversely JIA was more likely to be overlooked if the child had c*omorbidity such as learning disability or a chronic illness*.

**Conclusions:**

Care pathways are often ‘turbulent’ prior to a diagnosis of JIA with physical and emotional distress for families. There is need for greater awareness about JIA amongst health care professionals and observations of change (from family and non-health care professionals such as teachers) are key to trigger referral for paediatric rheumatology opinion.

## Background

Juvenile idiopathic arthritis (JIA) is a heterogeneous group of diseases with a spectrum of clinical features, disease course and prognosis [1]. It is one of the most common chronic inflammatory conditions in children and young people (CYP) with a prevalence of at least 1 in 1000 [2]. JIA is typically a treatable condition with optimal outcomes associated with early diagnosis and access to appropriate care [1, 3–5]. Delay has considerable impact on quality of life with pain, fatigue, and ultimately joint damage with functional disability; there is also potential for extra-articular complications including visual loss from JIA associated uveitis as well as high morbidity and potential mortality from macrophage activation syndrome [1, 5–7].

Prompt referral to an experienced paediatric rheumatology multidisciplinary team (PRh MDT) is recommended as soon as the diagnosis is suspected and in the UK the target is for the first assessment within 4 weeks of referral and 10 weeks from the initial onset of symptoms [8]. Unfortunately, for many CYP with incident JIA, delay in diagnosis and access to appropriate treatment remains a common problem reported around the world [3, 4, 9–12] and is a source of frustration and anxiety for parents and CYP [13, 14]. The reasons for delay are likely to be multi-factorial [3, 4, 15, 16], can occur at any point in the patient journey and may be influenced by a number of contextual factors [3]: *patient and family factors* (e.g. age, symptoms, carer concerns), *clinician factors* (e.g. previous experience, skills, knowledge) and *organisational factors* (e.g. availability of specialist advice, referral pathways, distance to specialist services and funding). These factors contribute to complex and potentially protracted care pathways to be negotiated by families between primary and specialist care [17] involving multiple healthcare visits [7] and investigations [6, 15].

The aim of this study was to identify, describe and understand determinants (barriers and drivers) to care in the context of incident JIA to inform strategies to address the challenges. Here we report findings of parental/guardians accounts of patients’ trajectories from initial symptoms to initial referral to paediatric rheumatology.

## Methods

We conducted in-depth qualitative interviews with the parent / guardian of CYP diagnosed with incident JIA presenting to a specialist regional paediatric rheumatology service at the Great North Children’s Hospital, Newcastle Hospitals NHS Foundation Trust, Newcastle upon Tyne, UK. Further participants from the families, health and non-health care professionals involved in the pathway of referral were recruited following the interview with the parent / guardian. Interviews where conducted face to face or over the telephone - with some interviews being joint interviews (with CYP, partner or other family member) - using topic guides and took place between November 2008 and July 2011. All participants were consented and older children (> 8 years) gave their assent to take part in the study. Interviewees did not receive payment. The study received a favourable ethical opinion from the Newcastle and North Tyneside 2 Research Ethics Committee (07/Q0906/59).

### Sampling strategy

Our sampling strategy was purposive to explore, test and refine emerging ideas to explore and map the diversity of factors that impact on delay and establish impact. We undertook four phases of data collection, recruiting different participants for each phase:

- *Typical case sampling* [18], *recruiting three groups of families: (i) Interval from reported symptom onset to diagnosis < 10 weeks; (ii) Interval 10–20 weeks; and (iii) interval > 1 year ‘protracted delay’. This approach was chosen to initially map some of the key issues.*
- *Criterion sampling* [18], *recruiting families who had received the diagnosis within the last 6 months. This enabled checking of emerging ideas and generating new areas to investigate.*
- *A further phase of criterion sampling, recruiting families who had received a diagnosis for over six months in order to validate our ideas with a different group of patients.*
- *Theoretical sampling* [18], *focusing on cases with very specific characteristics in order to test key aspects of our findings.*

A more detailed account of the sampling process and decisions has been outlined elsewhere [17] .

### Data analysis

Interviews were audio-recorded, transcribed and edited to ensure respondents anonymity. The initial topic guide evolved during the course of data collection, allowing for tailoring and gradual integration of a variety of follow-up issues and topics. All analysis was initially conducted by one of us (TR) according to the standard procedures of rigorous qualitative analysis [19]. We used procedures from first-generation grounded theory - coding, constant comparison, memoing [20] - and from analytic induction, deviant case analysis [21]. Sampling, data collection and analysis occurred concurrently, so that issues raised in earlier phases of fieldwork were explored subsequently to enable conceptual saturation [22]. We ceased sampling new cases when we deemed that we had achieved both ‘code’ and ‘meaning’ saturation [22] and that additional recruits would offer limited ability to extend ‘conceptual depth’ [23]. We also undertook regular data sessions (joint analysis meetings) where the research team share and exchange interpretations of key issues emerging from the data. The research team included social scientists and a paediatric rheumatologist (HF). We did not use any qualitative data analysis software to assist the formal analysis.

## Results

### Participants

We recruited 37 families - with one withdrawing from the study prior to interview – and undertook interviews to explore care pathways of 36 CYP with JIA. Patient details are described in Table 1. The median age was 6 years old (range 1–17), with a spread of JIA subtypes: (oligoarticular *n* = 14; polyarticular *n* = 16; systemic *n* = 3; psoriatic *n* = 3). The median reported time to first PRh MDT visit from symptom onset was 22 weeks (range 4-364 weeks). Interview participants included mothers (*n* = 34), fathers (n = 9), patients (n = 5), grandmothers (*n* = 2) and an aunt (*n* = 1). We also conducted 11 interviews with professionals: orthopaedic surgeons (*n* = 4), paediatricians (*n* = 3), paediatric immunologist (n = 1), General Practitioner [GP] (*n* = 1) nurse (*n* = 1) and primary school teacher (*n* = 1).

**Table 1:** Patient characteristics (parent/guardian-reported at time of interview)

ID	Age	Gender	JIA subtype	Time to first seeking medical advice from symptom onset (weeks)	Service symptoms first discussed in	Contacts with primary care centres prior to first referral to secondary care	Contacts with primary and secondary care centres prior to access to a PRh MDT	Referred to PRh MDT by	Time to first PRh MDT visit from symptom onset (weeks)
1	3	F	Systemic	> 1	A&E	1	13	Paediatrics	22
2	12	M	Oligo	1	GP	1	6	GP	364^a^
3	8	F	Systemic	> 1	GP	4	8	Paediatrics	9
5	7	M	Poly	8	A&E	1	5	Orthopedics	4
6	1	M	Oligo	> 1	GP-Out of Hours	2	13	Paediatrics	4
7	4	F	Poly	1	GP	1	5	Orthopedics	20
8	11	F	Oligo	4	GP	1	5	Orthopedics	26
9	5	F	Oligo	> 1	A&E	1	4	Orthopedics	26
10	5	M	Oligo	2	GP	1	7	GP	13
11	2	M	Systemic	> 1	GP-Out of Hours	4	8	Paediatrics	10
12	2	M	Oligo	4	GP	1	13	Orthopedics	8
13	3	F	Oligo	4	GP-Out of Hours	1	11	Paediatrics	9
14	6	F	Oligo	10	Health Visitor	4	9	Unclear	26
15	6	F	Oligo	5	Walk in Centre	1	4	Paediatrics	8
16	8	F	Poly	6	GP	3	3	GP	30
17	12	M	Poly	3	GP	2	2	GP	286^a^
18	6	M	Poly	4	GP	2	2	GP	7
19	16	F	Psoriatic	8	GP	1	2	Dermatology	52
20	4	M	Poly	> 1	A&E	2	17	GP	17
21	17	F	Poly	Unclear	GP	1	10	GP	20
22	13	F	Poly	14	GP	1	2	Rheumatology	22
23	11	F	Psoriatic	1	GP	6	6	GP	13
24	6	F	Poly	8	GP	4	5	Paediatrics	26
25	13	F	Poly	10	GP	1	3	GP	26
26	13	F	Poly	Unclear	GP	2	5	A&E	260^a^
27	12	M	Oligo	1	GP	1	2	GP	6
28	9	M	Oligo	> 1	GP	8	9	Orthopedics	32
29	7	M	Oligo	> 1	A&E	3	4	Orthopedics	13
30	17	M	Poly	N/A	Secondary (Paediatrics)	N/A	3	Paediatrics	104
31	3	F	Oligo	32	Health Visitor	10	16	A&E	52
32	5	F	Oligo	1	GP	1	1	GP	4
33	5	F	Poly	52	GP	4	12	Paediatric Immunology	104
34	3	F	Psoriatic	4	GP	2	4	Paediatrics	16
35	11	F	Poly	N/A	Dental	N/A	10	Paediatrics	208
36	5	F	Poly	4	GP	2	5	Paediatrics	22
37	5	M	Poly	12	GP	3	8	Paediatrics	77

^a^Participants received diagnosis prior – Patient 2 at 16 weeks, Patient 17 at 10 weeks and Patient 26 at 130 weeks - but did not visit PRh MDT care until this time point

### Determinants influencing diagnosis and referral

Overall, three factors emerged as being central to the pathways to appropriate care.

#### Persistence of symptoms

Parents reported either witnessing a change in their child (e.g. *observation of limp or joint swelling or change in mood or functional ability)* or their child complaining (e.g. *pain*) albeit these complaints could be vague, especially in the younger children. In some cases the first person to notice changes may not be the parent: a primary school teacher, a dentist, or other family member looking after the child when symptoms first emerged. Parents initially seek a ‘candidate explanation’ of the initial sign(s) and in so doing they understand them as tied to one of the following factors:

- *activities that the child has been undertaking (e.g. playing sport or related to a fall).*
- *development of the child (e.g. ascribing symptoms as ‘growing pains’).*
- *‘material’ environment (e.g. shoes too small or not fitting properly).*
- *child’s temperament (e.g. ‘being awkward’, or ‘school avoidance’).*

Families have various courses of action open to them. They may take no action as they rationalise the situation as ‘unproblematic’, for example, by assuming that they will ‘grow out of it’. They may also take a specific action:

- *Apply some form of remedy, such as buying new shoes or giving the child ‘over the counter’ medication.*
- *Seek more information. They may actively observe the child’s joint or movements for a brief or extended period looking for more evidence, or seek more details about the problem from the child including whether the child even feels it is a ‘problem’ for them, or adopt a ‘wait and see’ approach.*
- *Seek advice and support from others. These others include partner, family members or friends alongside using resources such as the internet. In only five of the 36 cases, did they seek advice from medical professionals within 24 h.*

In 18 cases, there was an interval between 1 week and 2 months for an initial visit to a medical professional. In two further cases the interval was 8 months and 12 months and parents felt the initial features were related to development of the child - the child had ‘funny square knees’ and the other child had stiffness related to her walking at a very young age and that her ‘muscles are developing’. So prior to the initial visit to a health professional parents engaged in a cycle of noticing, constructing an explanation and taking an appropriate action (see Table 2).

**Table 2:** Persistence of symptoms – examples of families accounts

**Families developed ‘candidate explanations’**	
Prior to an initial visit to health professionals, families reported engaging in cycles of noticing, constructing an explanation and taking action	Middle of January he started complaining about his legs hurting, well his leg in-particular but there was nothing to see so it was just, we were just kind of brushing it off as growing pains and whatever else and we thought it was a reaction, because [his] sibling had an operation we thought maybe he wanted a bit of attention and then there were one day when he was playing up with going to school and stuff like that “I don’t want to go to school, I don’t want to go to school” and then one day just out of the blue I was whipping his pyjama bottoms off to get him changed for school I was like “Oh blimey your knee it is really swollen up” … this was about 2 weeks I think of complaining his leg was swollen, sore, before we could actually see anything. … Yeah I even bought him some cream, some normal body lotion and said “Oh this is some magic cream it’s going to make you better”. And I think it was literally the next day his knee swelled up and I thought “Oh NO here we are brushing it to one side and there is something really wrong”. (Mother: P10, age 5–13 weeks to first PRh MDT visit, Oligoarticular^a^)
**No-real-concern-at-this-point trajectory**	
On visiting a health professional, some families reported they were told further investigation is not needed at this time	He just woke up one morning with a big swollen arm and my daughter thought he’d fallen out the bed or something and he she took him to the doctors [GP] and all they said they kept saying “it was just inflamed” and they were just giving him loads of different anti-inflammatory and things … She was at the doctors [GP] every week, 8 weeks it was … They were just saying “that [his arm] was inflamed, he might have knocked it” or whatever but then because he couldn’t use it, it just went stiff and it was all swollen … it was a case of “Oh he’s banged it” and “It’s just inflammation” they put him on Ibuprofen and calpol and by the time he did get here [PRh MDT] he couldn’t move. (Grandmother: P28, age 9–32 weeks to first PRh MDT visit, Oligoarticular)
**Further-investigation-is-required trajectory**	
As symptoms continue, escalate, or increase parents engage in repeat visits and referrals to both primary and secondary care	… he walked round ‘funny’ for a day, he walked round with his neck back so we got them to check it out … so we took him straight to [Out of Hours Service] they checked him out … and then a few days later his neck was fine but then he was struggling with his arms … so again we ended up back at [Out of Hours Service] erm and again they checked him over and they just thought it was post virus stiffness erm but then on the day that I took him to Mum’s Group and “he was really not right and he wouldn’t stand up … so I took him straight away to the actual doctors … and he sent us straight away to casualty … straight away he was on calpol erm to get his fever down … and he had no rash at that point but then rashes over the next couple of days, they kept us in overnight to watch him didn’t they. … the doctors put that down to any kind of viral rash they weren’t sure at that point what it was erm … A lot of the doctors at the time were just saying “its post viral, he’s had a cold and this is the virus coming out” … So then over the next few weeks we were to-ing and fro-ing because he just wasn’t getting any better, they gave us erm open access to the hospital … They thought he might have had an infection in his joints, so they x-rayed him and scanned him … they just kept on saying “No its just part of his cold, he’s just got a virus and this is what part of it is and it’ll go” and all this kind of stuff. … And this, this carried on and we kept on going in and out so every couple of days and he didn’t seem to be getting any better erm the rashes were on his body still and they were coming round more of his joints like his elbows, his knees and erm basically parts of his legs, sometimes on his back as well erm and there was like slightly raised redness on there erm so again we went back in to hospital with him, we had the open access and they came back and virtually said to us, “Well look its this virus” (Mother & Father: P11, age 2–10 weeks to first PRh MDT visit, Systemic)

^a^Information about interviewee includes: (Interviewee type: Patient Number, Patient Age – Family member estimate of time from onset of symptoms to first visit to PRh MDT, Diagnosis)

Only when the severity of the same symptoms increased or new physical, behavioural or mood features emerged alongside the existing ones, or symptoms continued over a period of time that was regarded as ‘going on for too long’, did parents seek medical opinion. In five cases, the initial physical features triggered parents to seek medical advice within 24 h of noticing the problem. The trigger in these cases was tied to a combination of the following factors: clear change in the degree of joint swelling; the potential seriousness of the lay-diagnosis for the parents (concern about meningitis given joint pains and a stiff neck; concern about vision (given that the child reported sore eyes and blurred vision); and the prompting from others to seek medical help (grandparents, partner).

The parents sought advice from primary care services, including General Practitioners, Out of Hours services - by phone or visit - and Health Visitors, alongside Accident and Emergency (A&E) departments and ‘Walk in Centres’. On these first visits two trajectories emerge.

- The first is a *no-real-concern-at-this-point* trajectory. Medical practitioners offer analgesia (e.g. ‘paracetamol’), adopt a ‘wait and see’ attitude (e.g. ‘come back in two weeks if still a problem’), position the concern as either not a problem (e.g. ‘nothing to be worried about’), or a behavioural problem (e.g. ‘ sometimes [children] walk funny’) or a developmental problem that does not require further medical concern (e.g. ‘growing pains’). In offering a reason for the diagnosis, practitioners provide an account embedded in the same range of factors that parents work with – namely, that the problem is related to the child’s activities, development, the material environment or temperament. This pattern of multiple health care visits continues until parents are referred to secondary care (see Table 2). In the UK, secondary care (i.e. hospital-based services such as general paediatrics, orthopaedic surgery) as well as tertiary care (sub-specialist services, such as paediatric rheumatology) can only be accessed by referral from primary care services, A&E or other hospital specialists. In our study, the referral to secondary or tertiary care resulted after multiple (2->10) visits to primary care or A&E.
- The second trajectory is *further-investigation-is-required*. In this context, if the initial visit is to a GP, the parents are referred to secondary care for further diagnostic evaluation. Alternatively, if they initially accessed care via out-of-hours services or a walk-in-centre, they are instructed to consult their own GP about the problem. However, once they are initially referred to secondary (or tertiary) care, the same pattern occurs. This can take 1->10 visits to secondary care and in some cases tertiary care to receive a referral to PRh MDT and a diagnosis of JIA. Parents can be discharged from secondary care and then have to repeat the process, visiting primary care to enable a referral to secondary care.

As symptoms continue or escalate parents engage in repeat visits and referrals to both primary and secondary care (including as an in-patient, often in a general paediatric ward), to establish a diagnosis and instigate treatment to manage the child’s symptoms (see Table 2). Referral to secondary or tertiary care can be expedited when there is a flare in symptoms or systemic upset (e.g. fever) that coincides with a visit to a practitioner. However, referral is further delayed when the patient has existing significant co-morbidities (e.g. dental problem with a small jaw [likely due to complications of JIA]; learning disability [Downs syndrome]; another chronic illness [psoriasis], known behavioural issues), or the family is engaged with some aspect of health and social care services (e.g. a parent with mental health problems or the child is subject to a safeguarding concern / care order) – in such cases the features are ascribed to other co-morbidities and a physical cause overlooked. As a mother (of a child with Down’s syndrome associated arthritis) noted, ‘when they’ve got a diagnosis, everything has to be related [to that]’ (Mother: P30, age 17–104 weeks to first PRh MDT visit, Polyarticular). This is an example of anchoring bias [24] where initial diagnostic ideas are retained, despite new (disconfirming) evidence. In such cases, the search for a new diagnosis is delayed by inappropriately assuming that new symptoms are explained by existing problems.

#### Parental persistence

Over the course of seeking a diagnosis and appropriate care, parents initially trust the health professionals’ views and are willing to accept their clinical opinion. However, over time, doubts enter parents’ minds, as their child is ‘still unwell’ despite repeated consultations. Parents have to manage the tension between trust and questioning the (diagnostic) expertise of health professionals. In one case, the parents sought to manage this tension through an experiment. They had been repeatedly told by their GP that the child’s lack of mobility and desire to be carried was that she was ‘a little bit of a lazy child’; so they placed chocolate at one end of the room and the child ‘dragged herself towards it’ (see Table 3). Although a dramatic example, it echoes the experiences of parents, in that they can begin to doubt themselves, and the advice given to them resulting in loss of trust to make judgements about the health of their child.

**Table 3:** Parental Persistence – examples of families’ accounts

**Trusting health professionals views**	
Families have to manage the tension between trust and questioning the expertise of health professionals	For instance we’d even try things at home which you’d know every child would be fooled by so you’d sit her at the other end of the room “do you want this chocolate bar” and she’d drag herself across the floor and you’d think well no child is going to drag themselves for chocolate they’re going to be up and running for it, so I mean to me that’s a fool proof plan (Father: P33, age 5–104 weeks to first PRh MDT visit, Polyarticular)
**Managing your own feelings**	
On visiting a health professional, some families reported they felt they were perceived as overly ‘anxious’	[At A&E] she started to sort of come round by then, her finger had gone down a bit and I had sort of explained you know we have been having problems … this kid has been poorly since Boxing Day we need to do something. … while we were actually there she actually came back to life and she was running round like a lunatic again and you sort of think hang on a minute, they are looking at you as if to say neurotic mother (laughter). (Mother: P01, age 3–22 weeks to first PRh MDT visit, Systemic)
**Seeking second opinions**	
Some families explicitly worked to see different health professionals other saw different ones by chance	And then one day at A&E a doctor said “Are you happy with what I’ve said” and I went “Not really” … I was a bit, when they were trying to say like it was nothing basically I wasn’t too happy. … me Mam wasn’t happy and I think they could tell we weren’t happy, … they referred us to the A&E clinic so I had to come back and see a doctor in the A&E clinic, they still said there was nothing wrong with her so I still said I wasn’t happy so they said they’d refer her to a consultant, luckily the one in [local town] was on compassionate leave and they said I could hang on till they came back or come to the [other town] (Mother: P31, age 3–52 weeks to first PRh MDT visit, Oligoarticular). I think for the third or fourth time … we had another set of x-rays done erm and a very stroppy consultant telling us that it was “all in her head” erm but, you know, “for goodness sake there was nothing on the x-ray”. … [another consultant] decided that we could come back to the fracture clinic the next morning and they would plaster her wrist as a precaution. … So erm we were trying to negotiate with the sister on the plaster clinic to, can we come at a different time … I knew the lady she said “Give me a moment I’ll ring the consultant upstairs, the adult rheumatologist” and she said “You need to come with me he’s actually up on the children’s ward” because he did different clinics “He’s got both sets of x-rays up on his computer screen as we speak” (laughs) we went to see him … we went up and he had both sets of x-rays on his screen side by side and that was the very first time he said “She had arthritis” and he said he wanted to refer her to [a different] hospital (Mother: P21, age 17–20 weeks to first PRh MDT visit, Polyarticular)

Parents have to continue to search for a diagnosis and treatment despite often feeling that health professionals perceive them as overly ‘anxious’, ‘worried’, ‘pushy’ or ‘wasting’ (professionals) time. In extreme cases they may even overhear this or be told directly by a health professional. At times, this feeling of concern about being labelled is compounded by fluctuation in the child symptoms and presenting with a ‘well child’ when assessed by the health professional (see Table 3). In order to address the ‘well child’ problem, parents may plan visits in order to align with a flare in symptoms (e.g. in the mornings) in order to demonstrate to practitioners’ the ‘symptoms-in-action’.

Parents engage in repeat visits to primary and secondary care due to a powerful sense of concern that what is going on with their child is not ‘normal’. Over time, parents get frustrated with the lack of a convincing solution to their child problems. Some parents directly ask for a second (or even third) opinion. Some gain second opinions by chance (see Table 3). For example by seeking an appointment with their usual GP and being told they are away and so resulting in being assessed by a different GP. Some gain a second opinion by design, for example by requesting an appointment with a different partner in a GP practice, going straight to A&E, visiting a walk-in-centre, changing practice or waiting for their GP practice to close before contacting them and so getting access to their out-of-hours service. In two cases, parents used private medical care for the first time to expedite referral to specialist opinion.

In maximising the number of different practitioners that they visit, in timing visits to align with flares in symptoms, or in repeatedly visiting the same practitioner, they seek to maximise the chance of either receiving a solution or being referred to another who may be able to offer a solution. In two cases other life circumstances (family bereavement) resulted in the parents being not wholly focused on the escalation of their child symptoms. In another case, the child had co-morbidities, and the initial onset of problems with the child’s toes was the ‘least of our problems’ (Mother: P19, age 16–1 year to first PRh MDT visit, Polyarticular). Therefore, without consistent parental persistence, delay can be extended even further.

#### Experience and skills of Health Professionals

Parents reported frustration with the uncertainty of a diagnosis and multiple consultations with different health professionals. This pattern of clinical activity seemed to lead to frustration for health professionals (see Table 4). If they positioned the child as on the *no-real-concern-at-this-point* trajectory they seemed to be frustrated with the parents. If the child was on the *further-investigation-is-required* trajectory, they could become frustrated with “need to name” the disease.

**Table 4:** Experience and Skills of Health Professionals – examples of families and health professionals accounts

**Parental frustration**	
Families often reported being frustrated with the uncertainty around the diagnosis	We’ve been to [Hospital One] fracture clinic, we’ve been to see another specialist they just kept sending us to see different specialists, we’ve been to [Hospital Two] ward to see somebody … they just didn’t seem like they had a clue. … They were just passing her backwards and forwards, fair enough it is hard with kids … We were passed back and forwards and nobody had a clue what was going on erm and they were seeing her in this pain but they couldn’t do nothing for her (Mother: P14, age 6–26 weeks to first PRh MDT visit, Oligoarticular)
**Health Professional frustration**	
Some families reported how health professional also appeared to be frustrated	You get to the stage when you feel like a pest when you’re ringing … One of the nurses I actually heard her on the phone, I rang and my phone didn’t have much signal where we live and I’d rang her on a Friday night because his neck was bad so the nurse had put a doctor on the phone and I was saying he’s been discharged but he’s got open access he’s on diclofenac, his necks bad, he’s got a temperature again and she couldn’t hear us properly and I went I’ll ring back and she went “And I can’t be bothered with this, with her with this problem” on the end of the phone. (Mother: P20, age 4–17 weeks to first PRh MDT visit, Polyarticular)
**Differential diagnoses**	
Health professionals needed to exclude other diagnosis prior to referral	You see the child could have anything else which is acute in nature and you need to really rule that out before I refer it, so it is kind of bottom of the list that er a child will be suffering from inflammatory arthritis (Orthopedic Surgeon: P12, age 2–8 weeks to first PRh MDT visit, Oliogoarticular) Things clearly weren’t getting better so you know we were clearly having to start thinking about other diagnoses as being more likely. … It wasn’t that I saw [P06] and thought “Gosh this looks very much like so and so” I think it was more a process of, erm, quite simply you know from what you read and what you are taught from text books you have a particular expectation of what something like a reactive arthritis should do and erm if its not doing that then you have got to think about other diagnoses. Now obviously you know I have seen a lot more cases of reactive arthritis than I have of inflammatory arthritis and perhaps another way of putting it is maybe its more to do with the fact that it was ceasing to look like a reactive arthritis rather than it was reminiscent of a child with an inflammatory arthritis. (General Practitioner: P6, age 1–4 weeks to first PRh MDT visit, Oliogoarticular) Well it’s not a case of pushing it’s a case of she wasn’t listening. It was all Down syndrome in her eyes, it had to be something to do with the Downs and it wasn’t and because it, the stiffness was spreading in his neck and elbows and everything he was completely seized up! But it had to be Downs related. (Mother: P30, age 17–104 weeks to first PRh MDT visit, Polyarticular)

In four cases, in the initial or second presentation to health practitioners, JIA was considered as part of the differential and raised as a potential diagnosis with parents. All but one of these children were then referred directly to the paediatric rheumatology team and the other to an adult rheumatology service. In other cases, JIA might have been raised at some point along the child’s trajectory, but health professionals reported as excluding JIA as the child did not appear unwell, was not in pain and/or they expected blood tests be able to confirm the diagnosis. Additionally, in some cases when inflammation is considered they may initially diagnose ‘reactive arthritis’. With persistent symptoms, reactive arthritis is then excluded and the child is routinely referred to paediatric rheumatology (see Table 4). However, as outlined above, in the context of a child with significant co-morbidities (e.g. dental problem with a small jaw [likely due to complications of JIA]; learning disability [Downs syndrome]; another chronic illness [psoriasis], known behavioural issues), some health professionals reported reticence to revise a diagnosis even when patients’ symptoms continue over an extended period of time (see Table 4).

Certain factors emerged to promote a practitioner considering a proposed or tentative diagnosis of JIA. ‘Hands on’ experience about JIA can be related to past experiences, in relation to memorable lectures, periods of training, or working alongside personnel from a paediatric rheumatology service. Awareness can be increased where the practitioner has been involved in a prior encounter of a child with JIA and more so with a ‘protracted pathway’ of a child in the past. As one parent put it, ‘ … it’s just getting to see the right person at the right time isn’t it ?…’ (Mother – P31, age 3–52 weeks first PRh MDT visit, Oligoarticular). However, even when a diagnosis of JIA is given, three parents felt that their child was not treated optimally, as they were not referred to paediatric rheumatology services. In one case, the child was given a brief period of physiotherapy in adult services and discharged relatively quickly, according to the mother, ‘because of his age they didn’t want to put him through all of these tests’ (Mother - P02, age 12–364 weeks to first PRh MDT visit, Oligoarticular). Over time, as the disease continued to flare, they were then referred to paediatric rheumatology and treatment escalated rapidly.

## Discussion

Delay in access to PRh care is a widespread problem and remains so even when specialist services are available [4, 5]. The impact of delay is considerable and given the highly effective treatments available [1, 3, 23], it is more imperative than ever that efforts are made to facilitate earlier diagnosis and prompt referral to specialist care.

Our study adds novel insights into key, and often interlinked factors that influence pathways to PRh care. We have described these broadly under key categories of *persistence of symptoms, persistence of parents help-seeking actions and experience of health professionals*. The referral to PRh care is invariably made by a health care professional in primary or secondary care and likely influenced to some degree by each of these factors.

Our dataset, with one exception, suggests that care pathways are often turbulent in terms of the effort families have to undertake in terms of repeat visits, the range and number of alternative diagnoses suggested, treatments offered and investigations endured prior to a diagnosis of JIA. The process and uncertainty often incurs physical and emotional distress. The complexity and turbulence was apparent whether the child has their first assessment within the UK recommended time frame [8] of 10 weeks or longer. Given the distress that can emerge, there is a need for awareness across the PRh MDT of the potential emotional impact, as well as increased access to appropriate resources at the time of diagnosis.

Our research highlights that health practitioners who see children need the appropriate experience and skills to consider a diagnosis of JIA; this includes prior experience of a child with JIA or exposure in some form to the PRh care (in clinical practice or a teaching/training event). It is known that many clinicians to whom children may present, lack self-reported confidence in their MSK clinical examination skills [24] and many doctors in primary care have not had training in paediatrics [25]. This may well contribute to the reported delay for other significant MSK diseases in CYP such as Slipped Upper Femoral Epiphysis [26] and cancer affecting bone and joint [27].

Messages from our work focus on the need for greater awareness about JIA, knowledge and skills to assess children appropriately, knowing when to consider JIA as a diagnosis and when to refer. Education and skills training needs to be targeted at those who are most actively involved in the care pathways of children, namely general practitioners, paediatricians, Accident & Emergency practitioners, orthopaedic surgeons, school nurses and allied health professionals. The educational messages can actually be quite simple, such as ‘JIA is not rare’, ‘children with JIA often appear well’, ‘pain may not be verbalised’, ‘there is no diagnostic test’, ‘basic blood tests and radiographs can be normal’; and ‘rheumatoid factor is invariably negative’ [28]. Our work suggests that the ‘ideal time’ to educate practitioners is when they have been involved in the care pathway of a patient with JIA and especially if the patient journey has been protracted. Clearly how this education is delivered requires sensitivity and needs to focus on being instructive rather than being critical. It is also important to consider the role of other professionals that work regularly with children. Paediatric physiotherapists will often assess children in the community and can likely ‘flag’ a child with suspected MSK pathology including JIA – this concept is the basis for the RightPath model of triage in the community - [29]. Furthermore, teachers and nursery workers also have a key role to raise concerns about a child as they know the child well and can notice *changes* that may indicate inflammatory joint problems: in the child’s abilities (*e.g. handwriting, play, sport*), avoidance of activities that they find difficult (*e.g. avoiding sitting cross legged on the floor or holding a pencil in the other hand*). Teachers are often trusted by families and have an important role to support and empower families to seek health care even if they don’t necessarily know what the cause of the problem is.

Raising awareness in the general population that ‘*children do get arthritis too*!’ is key and initiatives led by parents and CYP themselves are powerful ways to reach a wider audience and empower other families to seek medical attention. The emergence of social media and the internet has greatly increased capacity to share patient stories at low cost and wide reach [30] and the WORD day global awareness of JIA campaign exemplifies the impact of parents and CYP working alongside as partners with health care professionals to have wide reach and gather momentum [14].

Our study has several limitations. First, it was conducted in one region in UK and pathways of referral may differ in other parts of the world, for example, direct access to paediatricians rather than via primary care. Nonetheless we suggest that our findings are relevant to services elsewhere especially given that delay in access to care is a widespread problem. The Interviews with families were subject to issues of recall as families were asked to comment on retrospective events, which may have been months to years after diagnosis. We did not interview PRh about their perspective on their patients’ delays in diagnosis and future work should explore their insights into the process. Finally, our data was collected nearly 10 years ago (data collection ended July 2011) and so elements of the findings may be seen as no longer relevant, given changes in care. However, we know that delays in diagnosis remain a major problem impacting on clinical care [12] and are a priority to address in many parts of the world [31]; we suggest that the factors identified are still central to influencing that delay.

## Conclusions

This study describes the often turbulent care pathways prior to a diagnosis of JIA. Three factors – *the persistence of symptoms, parental persistence and the experience of health professionals* - need to align for a child with incident JIA to receive a diagnosis and appropriate care. Like the board game, snakes and ladders, this alignment process often happens through moments of ‘luck’ and ‘chance’. Health practitioners who see children need the appropriate experience and skills to consider a diagnosis of JIA. Educational messages need to be pitched at the appropriate level of complexity and targeted to both the myriad of health and non-health professionals who may prompt the diagnosis and the referral process.

## Data Availability

Due to the potentially sensitive nature of the research, no additional data is available. Interviewees consented to anonymised, exemplar, extracts of the data to being shared.

## Abbreviations

A&E: Accident and Emergency
CYP: Children and Young People
GP: General Practitioner
JIA: Juvenile idiopathic arthritis
PRh MDT: Paediatric Rheumatology Multidisciplinary Team
